# Comprehensive dataset on ripening stages of strawberries and avocados: From unripe to rotten

**DOI:** 10.1016/j.dib.2025.111663

**Published:** 2025-05-14

**Authors:** Pooja Kamat, Harsh Chandekar, Lisanne Dlima, Shilpa Gite, Biswajeet Pradhan, Abdullah Alamri

**Affiliations:** a Symbiosis Institute of Technology, Symbiosis International University, Near Lupin Research Park, Gram: Lavale, Tal: Mulshi, Pune, Maharashtra 412115, India; b Symbiosis Centre for Applied Artificial Intelligence (SCAAI), Symbiosis Institute of Technology, Symbiosis International University, Near Lupin Research Park, Gram: Lavale, Tal: Mulshi, Pune, Maharashtra 412115, India; c Centre for Advanced Modelling and Geospatial Information Systems (CAMGIS), School of Civil and Environmental Engineering, Faculty of Engineering & IT, University of Technology Sydney, NSW 2007 Australia; d Department of Geology and Geophysics, College of Science, King Saud University, Riyadh, Saudi Arabia

**Keywords:** Strawberries, Avocados, Fruit maturity assessment, Precision agriculture

## Abstract

This paper presents a novel and innovative 14,630 fruit images dataset, consisting of 1333 original images and the remaining augmented images for strawberry and avocado fruits. The dataset records the growth of strawberries and avocados in four different stages: unripe, partially ripe, ripe, and rotten. Though the fruit ripening process is commonly known, a lack of systematic datasets to show the fruit changing from an unripe state to a rotting state was prevalent for the two fruits in question. Over two months, the dataset was collected through rigorous tracking to effectively provide a measure of each of the fruits' conditions. The fruits were obtained from Mahabaleshwar farms in Maharashtra, India, as well as from local markets in Maharashtra and Pune. The fruits were monitored continuously from the time of harvesting, and all observed changes were carefully recorded. The uniqueness of this dataset is that it covers both strawberries and avocados, which have different patterns of ripening and are highly commercially valuable. The images were annotated using the online annotation tool - makesense.ai, with a total of 1499 bounding boxes for each fruit. By encompassing these two diverse fruit types, the dataset provides a valuable resource for researchers, agriculturalists, and food scientists to investigate and compare the ripening behaviours of different fruit species.

## Graphical abstract

**Figure:**
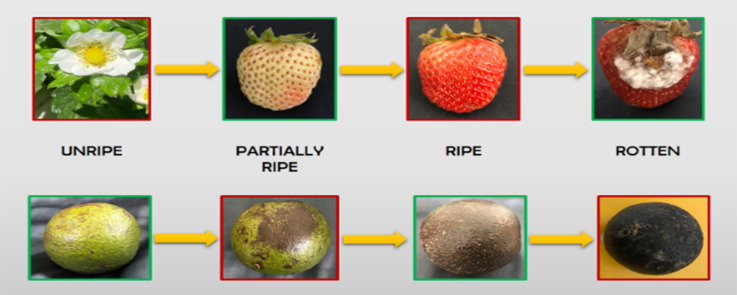

Specifications Table

**Table:** 

Subject	Computer Vision and Pattern Recognition
Specific subject area	Fruit Maturity Detection
Type of data	Raw images with label files in .txt format)
Data collection	Photographs were regularly clicked from the camera with dual 12 MP with f/1.6, 26 mm (wide) and 1.4 µm pixel size for 25 days by placing the fruit (subject) on it from different angles in bright light conditions. For the strawberries, pictures were taken daily, from the flowering stage until the fruit reached the rotten stage. The photographic documentation was carried out during the day with abundant natural light and clear visibility, ensuring optimal image quality. Each strawberry was captured from approximately three different angles to provide comprehensive visual information for analysis. In the case of avocados, raw fruits were purchased from the market, and a controlled environment was set up using a white background for enabling consistent and uniform image acquisition..
Data source location	Dataset was collected from (i) Mahabaleshwar, Maharashtra, India; and (ii) Pune, Maharashtra, India.
Data accessibility	Repository name: mendeley.com Data identification number: 10.17632/zysvgmxcyz.1 Direct URL to data: https://data.mendeley.com/datasets/zysvgmxcyz/1
Related research article	

## 1 Value of the Data


 

• This dataset is a useful resource for machine learning solutions in fruit maturity detection and can contribute to the design of automated sorting and classification systems by ripeness stages.
• Food processing companies and agricultural scientists may utilize this data to improve post-harvest management, packaging choice, and shelf-life estimation.
• The data enables research in precision agriculture by providing a labeled and organized sequence of images spanning the entire range of fruit ripening, from non-ripe to spoiled.
• Knowledge of ripening may assist in minimizing food loss and enhancing composting planning, towards achieving sustainable principles in food science and agriculture.
• This data can be utilized to create consumer advisory systems using AI to find the best ripeness level for consumption, thereby ensuring maximum nutritional value.

## 2 Background

The research seeks to offer a comprehensive dataset to trace the evolution of avocados and strawberries from their unripe phases to almost degradation through recording the physical changes and appearance of both the fruits at every ripening day through meticulous observation and the compilation of images on a daily basis [1]. The research intends to contribute to our understanding of the fruit's ripening dynamics. Ultimately, an increased understanding of fruit ripening will lead to improved models for ripening prediction, more effective post-harvest handling, and innovative fruit storage solutions for the forefront of modern food and agriculture [1]. With this dataset, researchers, horticultural professionals, and food scientists can study and compare how avocados and strawberries ripen, which should help advance our understanding of fruit ripening and also provide the necessary background to dig further into fruit physiology [2].

## 3 Data Description

**Fig. 1:**
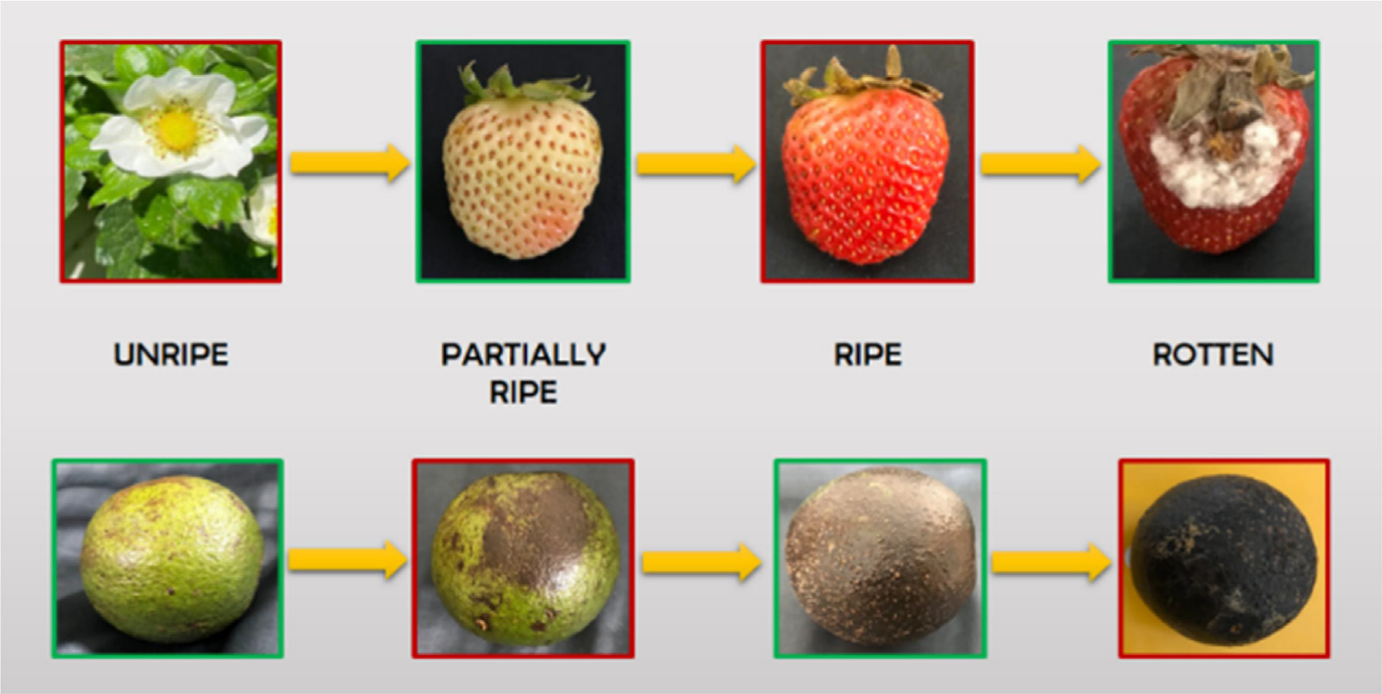
Sample images of strawberry and avocado in different stages.

Due to their considerable market value, the agriculture industry is particularly concerned with food waste, especially strawberries and avocados. Hence, these fruits were chosen for the dataset [3]. The dataset comprises 1333 images, each accompanied by corresponding text files and detailed bounding box information. The bounding box annotation uses the popular object-detection model YOLO (You-Only-Look-Once) [4]. The dataset includes four categories that describe the fruit’s growth stages: semi-ripe, ripe, rotting, and unripe, resulting in a total of eight categories (Fig. 1). The dataset is arranged methodically and divided into two folders: one containing the photos and another labelled for labelled data storage. File names in these folders start with IMG_0 and go up to IMG_1332, totalling 1333 records in each fruit case. Many photos contain multiple bounding boxes, significantly enhancing the dataset's effectiveness. Across all the image collections combined, there are 1499 bounding boxes. These bounding boxes are crucial as they enable accurate localisation and clear identification of the specific developmental stages displayed by the avocados and strawberries in the images. Emphasising the importance of these bounding boxes is paramount [5]. They provide the structural basis for careful examination and in-depth study of the dynamic processes involved in the progressive ripening of avocados and strawberries. Their addition makes it possible to analyse how these fruits change over time.

To offer a thorough summary, the paper presents a tabular representation below illustrating the prevalence of various developmental stages within the split image categories (Table 1).

**Table 1:** Category count of strawberries(S) and avocadoes (A).

Category	Count
SUnripe	114
SPripe	238
SRipe	189
SRotten	170
AUnripe	185
APripe	192
ARipe	129
ARotten	282

### 3.1 Dataset preprocessing

**Fig. 2:**
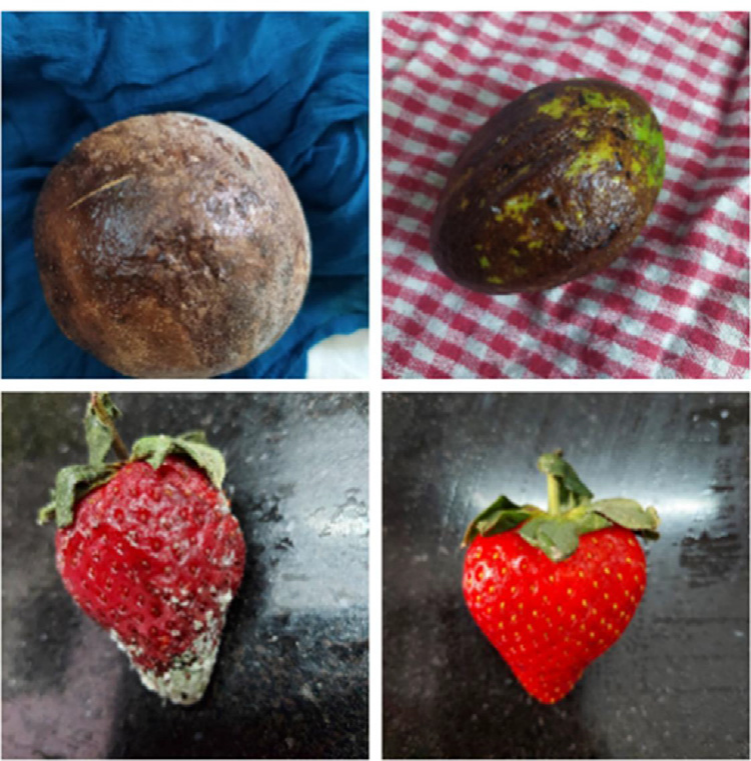
Images before background change.

**Fig. 3:**
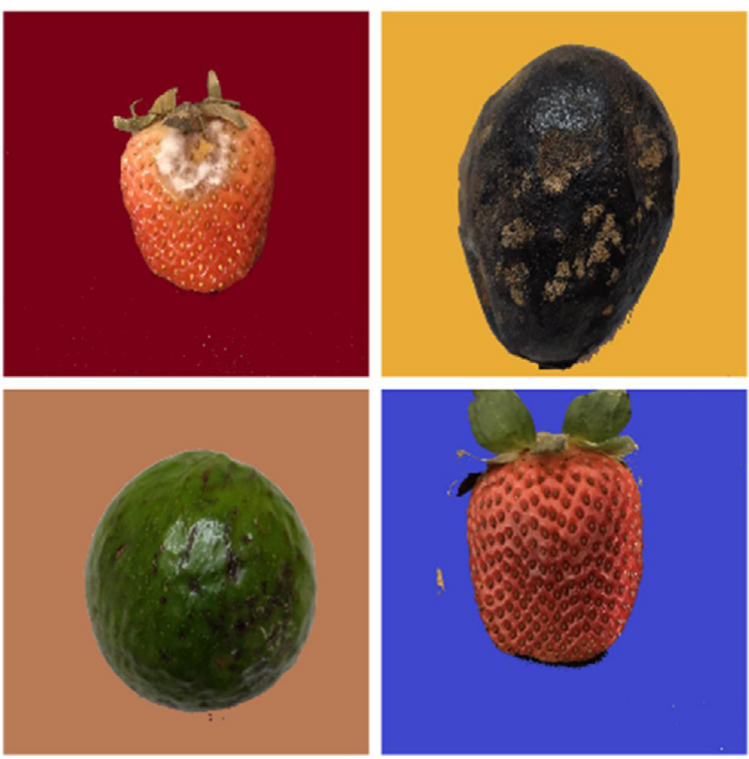
Images after background change.

As seen in Fig. 2, the dataset utilised in this investigation consisted primarily of photos taken against a uniform background, which could have introduced bias by allowing the model to rely on background signals for fruit classification. To mitigate this bias, an important pre-processing step was replacing each image's background with a solid pastel color. Adopting shades of red, green, blue, purple, orange, and yellow aimed to enhance accurate fruit identification and prediction by reducing the impact of background-related information displayed in Fig 3.

### 3.2 Dataset balancing

The dataset balancing process was conducted to ensure a balanced representation across fruit categories. A subset of 58 images, along with their corresponding bounding boxes, was selected for each category, resulting in a total of 409 photos, in the final dataset due to this rigorous curation. The balanced dataset is stored in two folders, one holding the labels and the other the photos. It is important to note that an equal distribution of instances was obtained for each fruit type following this stringent balancing technique. Table 2 shows the precise count for each category.

**Table 2:** Balanced count of Strawberry and Avocado samples across different ripening stages.

Category	Count
SUnripe	58
SPripe	58
SRipe	58
SRotten	58
AUnripe	58
APripe	58
ARipe	58
ARotten	58

## 4 Experimental Design, Materials and Methods

**Fig. 4:**
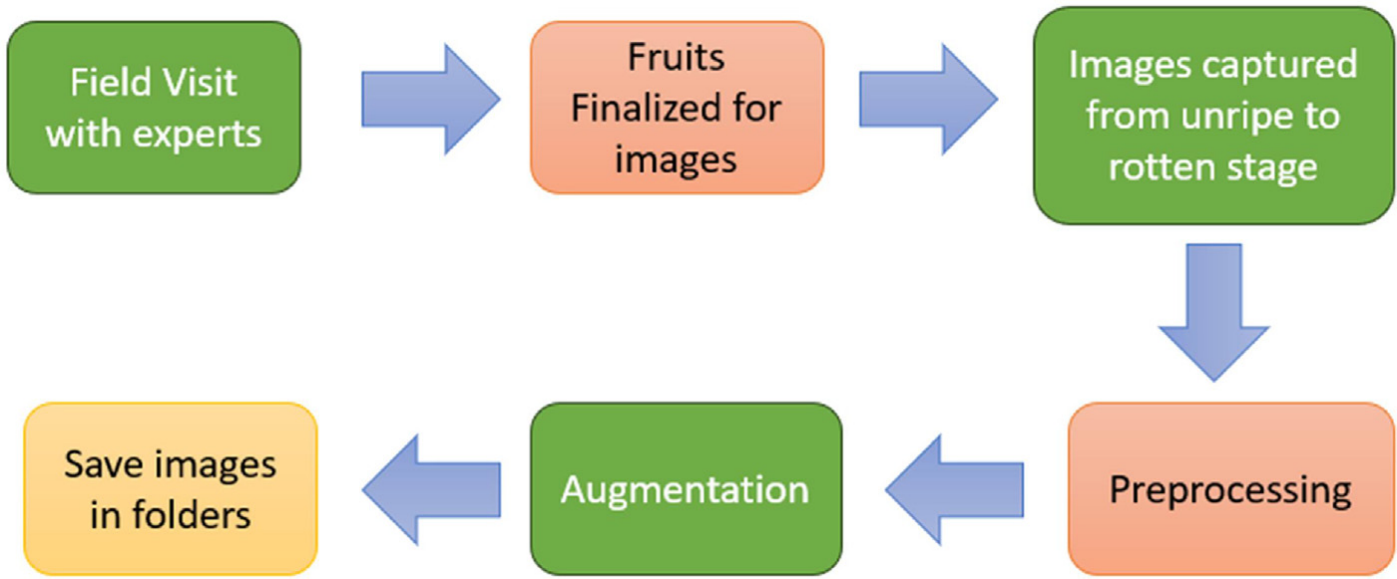
Process of dataset preparation.

The experiment began with a thorough study of fruit taxonomy and ripening stages based on literature [6] and field studies. The subject fruits were monitored from their first stages to defoliation. Photographs were then collected and arranged subsequently in files named IMG_0 –> IMG_1332. To facilitate model training and ensure color consistency across all images, the backgrounds were converted to pastel colors. Th images were manually annotated to provide ground truth bounding boxes, which are essential for any object detection algorithm to function properly [7]. Augmentation techniques such as rotating images, carefully controlled blurring, and zooming in/out were employed to enhance the dataset's comprehensiveness [8]. Further, this augmented dataset was then classified and assigned to systematic folders. Fig 4 shows the corresponding steps.

### 4.1 Sampling and collection

• Strawberries: The dataset includes Sweet Charlie and Winter Dawn varieties, sourced from farms in Mahabaleshwar, Maharashtra, India.
• Avocados: The dataset focuses on the Hass variety, sourced from local vendors in Pune, Maharashtra, India.
• Data collection was conducted over 25 days, with daily monitoring of each fruit’s ripening progression.

### 4.2 Environmental conditions

• Strawberries were monitored in-field under natural daylight conditions, capturing multiple angles for comprehensive analysis.
• Avocados were placed in a controlled indoor environment, ensuring uniform lighting conditions and background standardization.
• Replications were performed through continuous monitoring, ensuring that each stage of ripeness was well-documented.

### 4.3 Dataset annotations

There are a total of 4 categories for each fruit - unripe, partially ripe, ripe and rotten. The annotation tool makesense.ai was used to manually annotate all the images. Bounding boxes were created for annotation, and in some images, multiple bounding boxes were used due to the presence of several similar fruits within the same image frame. The images were then sequentially numbered and checked for any errors or anomalies.

### 4.4 Dataset export

Upon completion of the annotation task, the entire dataset was resized to a resolution of 1024 × 1024 pixels uniformly for the sake of data quality and consistency. The dataset has also been shared on the Mendeley repository so that it is easily accessible and usable for future research and development.

## Limitations

The dataset, while being comprehensive, has a few limitations as follows:

• **Limited Geographic Scope**: Fruits were only obtained from two locations (Mahabaleshwar and Pune, Maharashtra, India), and they might not reflect the ripening trends of strawberries and avocados in other locations.
• **Limited Temporal Coverage**: Samples were taken within a span of two months, which could potentially fail to pick up the full scope of ripening behaviours over varying environmental conditions..
• **Focus on Two Fruit Types**: Although valuable, this dataset only includes information on strawberries and avocados, which may limit its applicability to generalizing the ripening processes of other fruit species.
• **Potential Bias in Fruit Selection**: Fruits were purchased from farms and local markets, which could introduce variability in ripening stages due to differences in handling, storage, and transportation practices.
• **Small Number of Original Images**: The dataset contains only 1333 original images, with the remaining images being augmented versions, potentially limiting the diversity of the dataset.

By addressing these limitations through further dataset expansion, the concept of fruit maturity detection can be significantly enhanced, leading to improved performance and broader applicability in real-world applications.

## Ethics Statement

The authors have confirmed that this study does not involve human subjects, animal experiments, or any data collected from social media platforms and follows the ethical requirement for publication in Data in Brief.

## CRediT Author Statement

**Pooja Kamat:** Conceptualization, Investigation, Data curation, Writing – original draft, Visualization; **Harsh Chandekar**: Conceptualization, Investigation, Data curation, Writing – original draft, Visualization; **Lissane Dlima**: Conceptualization, Investigation, Data curation, Writing – original draft, Visualization; **Shilpa Gite:** Writing - Review & Editing, Supervision; **Biswajeet Pradhan:** Writing - Review & Editing, Supervision, Funding, Resources; **Abdullah Alamri:** Writing - Review & Editing.

## Data Availability

Mendeley DataComprehensive Dataset on Ripening Stages of Strawberries and Avocados: From Unripe to Rotten (Original data).
